# Editorial: Rehabilitation within the context of palliative care

**DOI:** 10.3389/fresc.2024.1490057

**Published:** 2024-09-18

**Authors:** Marcos Montagnini, Christopher M. Wilson

**Affiliations:** ^1^ Division of Geriatric and Palliative Medicine, University of Michigan, Ann Arbor, MI, United States; ^2^ Physical Therapy Program, Oakland University, Rochester, MI, United States; ^3^ Department of Physical Medicine and Rehabilitation, Corewell Health, Troy, MI, United States

**Keywords:** rehabilitation, physiotherapy, occupational therapy, speech-language pathology, physical and rehabilitation medicine, hospice, end-of-life, quality of life

**Editorial on the Research Topic**
Rehabilitation within the context of palliative care

The rapid global expansion of palliative care and associated positive patient care and cost savings outcomes have concurrently resulted in an increased demand for high-quality rehabilitation services with this patient population (1). Historically, rehabilitation professionals’ interventions have been commonly considered to be restorative as opposed to palliative, with an attempt to regain function and correct physical limitations (2). Yet, some of the earliest rehabilitation services have been with individuals with a life-limiting condition or chronic disability, including those wounded in World War I and the polio epidemic (2). Rehabilitation treatment plans were implemented to slow physical decline, optimize the patient's remaining quality of life (however long that may be), minimize suffering, and utilize their remaining physical capabilities to meaningfully engage with society to their utmost capacity (3). These palliative rehabilitation approaches remain relevant with recent events such as the COVID-19 pandemic or health innovations that have made life-threatening diseases like cancer and HIV more akin to chronic diseases to be managed.

Yet, despite the positive outcomes of palliative rehabilitation interventions, there remains sparse and inconsistent integration of rehabilitation into palliative care services and teams. Recognizing this critical gap, the World Health Organization (WHO) European Region published a *Policy brief on integrating rehabilitation into palliative care services* (4). This landmark document illuminates the critical role of rehabilitation for these patients and provides guidance on best practices for clinical integration and organizational sustainability. An essential action step from this document was to “Obtain additional evidence on rehabilitation models in palliative care…” To that end, we had the honor of editing this research topic entitled *Rehabilitation within the context of palliative care*. We are so thankful to the authors for providing their insights and efforts to advance the collective knowledge of this rapidly growing practice area. Each article selected for this research topic speaks to a unique component of the critical role of rehabilitation in this patient population and adds to the body of literature addressing the importance of rehabilitation for patients with life-limiting illnesses.

One of the most common rehabilitation interventions is exercise and physical activity, yet we know very little about patients’ lived experiences with exercise in palliative care. Young et al. utilized a meta-synthesis approach to assess for commonalities in the main findings from eight qualitative studies. Common themes included the impact of the delivery method of exercise, participants’ emerging motivation, and the physical effect of exercise. Participants reported that “Exercise was able to offer a sense of ‘orientation’ through providing purpose and coherence in everyday life at a time when this may have been lost due to the illness.” In concert with the myriad health and functional benefits, this finding provides evidence that exercise can provide the critical but elusive concept of meaning and purpose.

The WHO policy paper emphasized the importance of and unique challenges to integrating rehabilitation within palliative care services in low- and middle-income nations. Ogundunmade et al. were well positioned to share their perspective and experiences of the barriers and opportunities to this integration in developing countries. The authors highlighted the importance of therapists having a comprehensive understanding of the diseases and conditions that their population is facing and used the example of HIV/AIDS in sub-Saharan Africa as a population need that other global areas may not be struggling with. As with any other healthcare service, coordinated care and funding were critical issues that must be addressed to provide integrated, comprehensive rehabilitation and palliative care services.

Manzino and Wilson described a unique case of a hospitalized patient with progressive hip pain from metastatic breast cancer to the bone. In this case, the treating physical therapist continued to note worsening pain and debility and proactively advocated for appropriate imaging and orthopedic consultation. This resulted in a prophylactic hip fixation to prevent an imminent fracture. This case report highlighted the communication challenges and critical need for rehabilitation professionals to seamlessly integrate into the palliative care team to assure optimal outcomes and patient safety.

Preston et al. demonstrated that using trained volunteers to optimize the functional independence of individuals with advanced progressive illness receiving home hospice is feasible. Following an assessment by the therapist, patients were matched with a trained volunteer who supported 4–8 rehabilitation sessions in the person's home. This intervention improved mobility, general health, and achievement of goals. It was the first study demonstrating the benefits of using trained volunteers to support the rehabilitation of patients receiving hospice care. It represents a promising model of care for people living in rural and underserved communities.

Lastly, Aljassem et al. thoroughly describe the concept of palliative care and rehabilitation for patients with substance use disorders (SUD). Patients with longstanding SUD represent a unique challenge for healthcare professionals due to its chronic and relapsing nature, high morbidity and mortality due to organ failure, chronic infections, and overdose syndromes. The integration of palliative care into the care of patients with SUD is strongly desirable because of the unique characteristics of this population. The authors recommend an increased emphasis on engaging palliative care in SUD management and proactive integration of rehabilitation services into the palliative care team to provide a comprehensive and patient-centric approach that can substantially impact the patient's well-being and quality of life.

The topic of *Rehabilitation within the context of palliative care* published by *Frontiers in rehabilitation sciences* adds to the body of evidence demonstrating the benefits of rehabilitation for patients with serious and life-threatening diseases. However, more research is needed to advance the field so that patients and their families can benefit from such approaches. While the field of palliative rehabilitation for cancer populations has progressed steadily, rehabilitation for patients with other diseases still needs to advance. Studies addressing the benefits of rehabilitation for patients with progressive neurological disorders, severely disabling neurological or traumatic conditions (e.g., locked-in syndrome, traumatic brain injury, tetraplegia), congestive heart failure, chronic lung disease, and organ failure are necessary as the population living with these conditions continues to expand worldwide. Further investigations are needed to identify predictors of functional improvement as well as effective pain management, and psychological distress from rehabilitation interventions with chronic or life-limiting conditions. Investigations and quality improvement projects are needed to establish the various resources needed to deliver palliative and rehabilitation services (particularly in low- and middle-income countries) including types of interventions and proper settings in which to deliver these interventions. It is also important to educate professionals dealing with patients with progressive and life-limiting illnesses about the need for and benefit of rehabilitation, so that services and support can continue to be available for these patients. Conversely, rehabilitation professionals should strive to educate their rehabilitation colleagues on the role and importance of palliative care during rehabilitation. These key steps can help to ensure that every patient facing a chronic or life-threatening illness has unfettered, equitable access to palliative care and rehabilitation to help them pursue optimal quality of life and dignity.
